# Fractional CO_2_ Laser to Treat Surgical Scars: A System Review and Meta‐Analysis on Optimal Timing

**DOI:** 10.1111/jocd.16708

**Published:** 2025-01-08

**Authors:** Qiang Ji, Lili Luo, Jun Ni, Xiaolan Pu, He Qiu, Dongmei Wu

**Affiliations:** ^1^ Department of Aesthetic Plastic Surgery, West China School of Public Health and West China Fourth Hospital Sichuan University Chengdu China; ^2^ Department of Anesthesiology, West China Hospital Sichuan University Chengdu China

**Keywords:** fractional carbon dioxide laser, scars, surgery

## Abstract

**Background:**

Surgical scars with textural changes can be disfiguring and uncomfortable for patients. Various laser therapies have shown promise in softening and flattening these scars. Therefore, the authors conducted a systematic review and meta‐analysis on the efficacy of fractional CO_2_ laser in treating surgical scars.

**Objective:**

This study aims to present evidence from controlled trials investigating the efficacy of ablative carbon dioxide fractional laser in treating surgical scars.

**Materials and Methods:**

A literature search of Medline (via PubMed), Ovid, Web of Science, and Embase for relevant trials was conducted before March 2024. After assessing for inclusion, data extraction was performed using Population, Intervention, Comparison, Outcomes and Study criteria (PICOS). Quality, validity, and risk of bias were assessed using the RevMan5.3 risk of bias assessment tool.

**Results:**

A total of 14 controlled trials involving 492 participants or postsurgical scars were included in the system review and meta‐analysis. Both in RCT and non‐RCT settings, fractional CO_2_ laser therapy exhibited the same efficacious outcomes, with MD values of −0.63 (95% CI: −1.15 to −0.12; I^2^ = 70%; *p* = 0.02) and − 1.86 (95% CI: −2.65 to −1.07; I^2^ = 85%; *p* < 0.001), respectively. Moreover, furthermore analysis illustrated that initiating FRACTIONAL CO2 LASER treatment sessions at or within 1 month after surgery significantly reduced postoperative scars compared to control groups and groups on treatments initiated more than 3 months after surgery (MD: ‐1.66; 95% CI: −2.31 to −1.01; I^2^ = 89%; *p* < 0.001 and MD: ‐1.93; 95% CI: −2.24 to −1.62; I^2^ = 48%; *p* < 0.001). However, fractional CO_2_ laser treatment administered over 3 months after surgery did not significantly improve postoperative scars (MD: –0.17; 95% CI: −0.56 to 0.21; I^2^ = 37%; *p* = 0.37).

**Conclusion:**

The systematic review and meta‐analysis provide robust support for the efficacy of fractional CO_2_ laser in treating surgical scars, particularly when administered at or within 1 month after surgery. One treatment session within 1 month after surgery also can produce significant results, but most clinical trials support 2–3 treatment sessions or more.

## 1 Introduction

Surgical scars represent a natural part of the wound healing process, where fibrous tissue replaces normal skin following injury [1, 2]. While many scars resolve over time, some can manifest as hypertrophic or keloid scars, causing discomfort and disfigurement for patients [3, 4]. Laser therapies, notably fractional CO_2_ laser treatments, have emerged as promising interventions to soften and flatten these scars, offering relief from symptoms such as pain and itching [5, 6]. The efficacy of fractional CO_2_ laser in treating various types of scars, including burns and acne scars, has been well‐documented [7]. However, the application of fractional CO_2_ laser therapy specifically for surgical scars remains a topic of debate, with conflicting findings reported in the literature. Notably, studies have varied in their timing of fractional CO_2_ laser treatment, some targeting mature scars while others focusing on scars in the early postoperative period [8, 9, 10, 11, 12].

Complicating matters further are discrepancies in scar assessment methodologies, with different scoring systems yielding divergent results [13]. Many trials suggest fractional CO_2_ laser intervention on surgical wounds achieves favorable outcomes with VSS scores. There are also some trials that showed (observer) and objective measurements in terms of PhGA or POSAS could not confirm its efficacy in the treatment of surgical scars [14]. Considering these uncertainties, our systematic review aims to address the crucial question of optimal timing for fractional CO_2_ laser treatment in surgical scars. By synthesizing data from prospective controlled trials, we seek to elucidate the effectiveness of fractional CO_2_ laser interventions at different stages of scar maturation. Specifically, we aim to assess outcomes related to treatment sessions were, while considering variations in study methodologies and assessment criteria.

Through our comprehensive analysis, we aim to provide evidence‐based recommendations to guide clinical practice and optimize treatment outcomes for patients with surgical scars. By shedding light on the role of timing in fractional CO_2_ laser therapy, we endeavor to enhance the efficacy and accessibility of this intervention, ultimately improving patient care and quality of life.

## 2 Data and Methods

### 2.1 Search Strategies

A literature search of Medline (via PubMed), Ovid, Web of Science, Embase, and Cochrane for relevant trials was performed before March 2024. Relevant keywords and MeSH terms were used in logical combinations: (cicatrix OR scar OR scars) AND (10 600 laser OR Carbon Dioxide laser OR CO_2_ laser) AND (treatment OR therapy) AND (surgery OR surgical OR postoperative).

### 2.2 Inclusion Criteria and Exclusion Criteria

Inclusion criteria were as follows: 1. The trials were prospective controlled trials. 2. The study was published in English. 3. The experimental group was treated with a fractional CO_2_ laser. 4. The control group had untreated scars, or the scar treatment measures of the experimental group after fractional CO_2_ laser treatment were consistent with those of the control group. 5. Treatment scores were: change (a) Vancouver Scar Scale (VSS); (b) Patient and Observer Scar Assessment Scale (POSAS); (c) Modified Manchester Scar Scale (MMSS).

Excluded criteria included the following: 1. Treatment of acne scars, burn scars, tattoo, and port wine stains. 2. The studies were case–control studies and review studies. 3 The studies were history of chemical peeling and other previous laser or resurfacing procedures to the scar. 4. fractional CO_2_ laser and other laser comparative experimental studies.

### 2.3 Assessment and Clinical Data

Assessment for quality, validity, and risk of bias applied a scale devised by the RevMan risk of bias assessment tool. Two investigators assessed the methodological quality and risk of bias of the included studies independently according to the Cochrane Handbook for Systematic Reviews (http://www.cochranelibrary.com/), which consists of seven items:(1) random sequence generation, (2) allocation concealment. (3) blinding of participants and personnel, (4) blinding of outcome assessment, (5) incomplete outcome data, (6) selective reporting, (7) and other biases. Disagreements were resolved through discussion.

### 2.4 Analysis and Methods

We used Revman5.3 software for meta‐analysis. For continuous data, if standardized mean difference (SMD) is used for analysis: the relative risk RD value is calculated for the two categories of data, 95% CI is calculated for all the analyses, and the heterogeneity among the studies is determined by the Cochrane *Q* test. If there was no statistical heterogeneity (*p* > 0.1 I^2^ < 50%) between the studies, the fixed effect model was used; if there was heterogeneity (*p* < 0.01 I^2^ ≥ 50%) between the studies, but there was no clinical heterogeneity, the random effect model was selected. If the source of heterogeneity cannot be determined, descriptive analysis is used.

## 3 Results

### 3.1 Analysis of Basic Information

**FIGURE 1:**
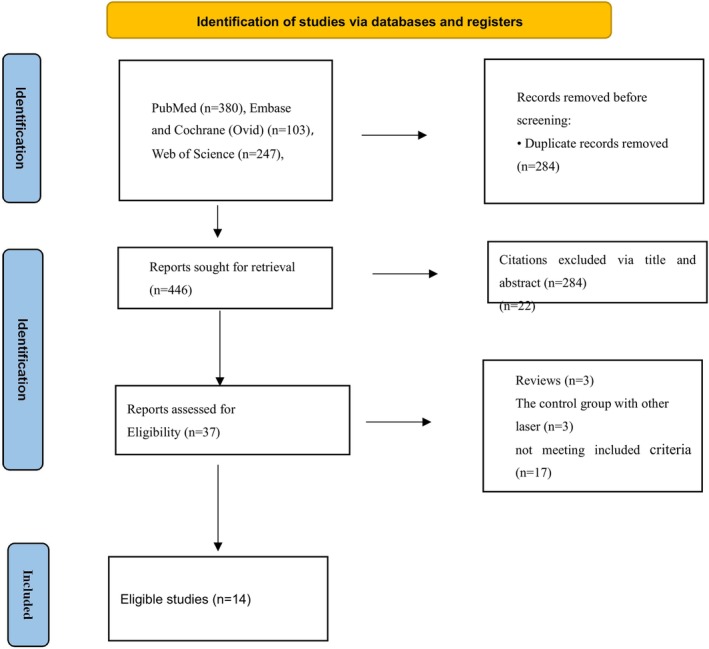
Flowchart of the study selection process.

Figure 1 presents the details of the study selection process. Our initial search retrieved 730 citations. Among these citations, 37 articles were considered potentially eligible after scanning the titles and abstracts. In total, 14 were included in this systematic review met our inclusion criteria, after reading the full texts on fractional CO_2_ laser treatments. Meta‐analysis was performed with available and comparable data.

### 3.2 Basic Features of the Included Literature

(Table 1) A total of 14 controlled trials involving 492 participants or postsurgical scars were included in the system review and meta‐analysis. Six trials were performed in Asian countries, 3 in Korea and 3 in China, 4 trials in Europe and America, 2 in Egypt, 1 in Iran, 1in Brazil. Fourteen trials included 270 scars. Nine were RCT and 5 were non‐RCT. Among these, 12 studies focused on VSS and included 457 postoperative scars undergoing fractional CO_2_ laser treatment sessions for meta‐analysis, 2 applied POSAS, and 1 MMSS. In the meta‐analysis, 9 treatment trials completed within 1 month after surgery, and 3 were performed for scars over 3 months after surgery, 4 indicated that only one fractional CO_2_ laser treatment was performed during the follow‐up period. One article on the comparison of laser energy and 1 article on the comparison of laser treatment time.

**TABLE 1:** Clinical data included in this study.

Study ID	Country	Study type	Anatomic areas	Fitzpatrick skin type	Participants	Design and setting	Intervention	Control	Starting time	Session	Outcome indicator
Lee, S.H [15] 2013	Korea	Non‐RCT	Face, arm, neck, back and chest		15 M/F: 8/7	Split scar	10 600 nm fractional CO_2_ laser	Untreated	3 weeks after surgery	2 s/3 m	VSS
Sobanko. J. F [16]. 2015	American	RCT	Face	I or II	20 M/F: 15/5	Split scar	10 600 nm fractional CO_2_ laser	Untreated	Day of suture removal	1 s/ 3 m	VSS
Buelens, S. [14] 2017	Belgium	RCT	Head and neck		9 M/F: 3/6	Split scar	10 600 nm fractional CO_2_ laser	Untreated	In 3 months after surgery	3 s/3 m	PhGA, PGA, POSAS
Karmisholt, K. E. [17] 2017	Denmark	RCT	Caesarean		11 F	Split scar	10 600 nm fractional CO_2_ laser	Untreated	More than 1 year after surgery	3 s/6 m	POSAS VSS
Alberti, L. R. [18] 2017	Brazil	RCT	Breast and abdomen	I–IV	41F	Non‐split scar	10 600 nm fractional CO_2_ laser and silicone gel	silicone gel	3 weeks after surgery	(a single session) /6 m	VSS
Darwish, A. M. [19] 2017	Egypt	Non‐RCT	Face	III–V	20 M/F: 11/8	Split scar	10 600 nm fractional CO_2_ laser	Untreated	2–3 weeks after surgery	6 s/3 m	VSS
Zhang, Y. S. [20] 2020	China	Non‐RCT	Face and neck	III–V	33 M/F: 11/22	Non‐split scar	10 600 nm fractional CO_2_ laser	Untreated	Within 1 week after suture removal	1–3 s/9 m	VSS
You, H, J. [21] 2020	Korea	Non‐RCT	Neck		24 M/F: 4/20	Split scar	10 600 nm fractional CO_2_ laser	Untreated	1 months after surgery	5 s/6 m	VSS PGA
Mohamed, S. [22] 2020	Egypt	Non‐RCT	Cleft Lip		120 M/F: 4/20	Non‐split scar	10 600 nm fractional CO_2_ laser	local creams	3 weeks and 3 months after surgery	5‐7S/6 m	VSS
Shin, H. W. [23] 2021	Korea	RCT	Breast		15 F	Split scar	10 600 nm fractional CO_2_ laser	Untreated	day of suture removal	a single session	VSS and VAS
Fallahi, H. R. [24] 2021	Iran	RCT	Nose	III–IV	26 M/F: 1/25	Non‐split scar	10 600 nm fractional CO_2_ laser	Untreated	4 months after surgery	a single session	VSS
Chi, H. [25] 2023	China	RCT	Cleft lip	III–V	42		10 600 nm fractional CO_2_ laser 1‐month group, 3‐month group, 6‐month group after surgery	Untreated	1‐months, 3‐months, 6‐months after surgery	3 s/3 m	VSS
Wang, J. [26] 2023	China	RCT	Periorbital	III–V	90 M/F: 36/54	Split scar	10 600 nm fractional CO_2_ laser (High fluences with low‐density treatment group)	10 600 nm fractional CO_2_ laser (Low fluences with low‐density treatment group)	2 weeks after surgery	4 sessions with a 4‐week interval	VSS
Lin, M. J. [27]	American	RCT	Skin Cancer	I or II	26 M/F: 15/11	Split scar	10 600 nm fractional CO_2_ laser 0‐Day group	10 600 nm fractional CO_2_ laser 14‐Day group	0‐Day and 14‐Day after Surgery	a single session	MMSS

*Note:* AFL, fractional CO_2_ laser. M/F, man, and female; untreated, placebo treatment in the control group. Study ID, last name of the first author and year of publication, last name of the investigator.

Abbreviations: MMSS, Modified Manchester Scar Scale; PGA, patient global assessment; PhGA, physician global assessment; POSAS, Patient and Observer Scar Assessment Scales; VAS, visual analog scale of cosmetic appearance; VSS, Vancouver Scar Scale.

### 3.3 Characteristics and Risk of Bias of Included Studies

**FIGURE 2:**
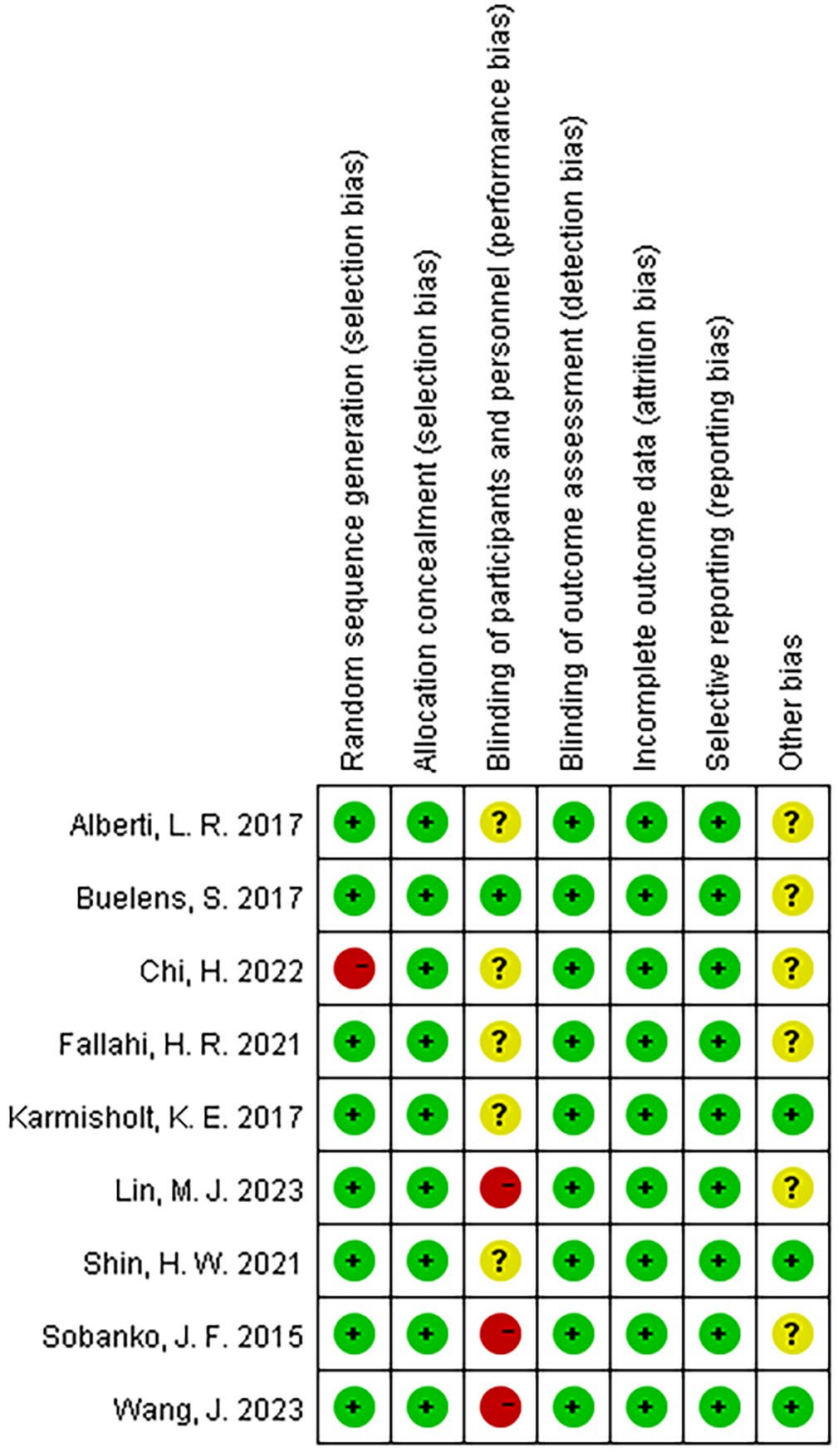
Risk of bias summary.

According to the RCT quality assessment, the risks of bias regarding randomization allocation concealment, and the risk of bias for blinding of outcome data were high. However blinding of participants and personnel was low. Other bias were unclear (Figure 2).

### 3.4 Meta‐Analysis Results on Fractional CO_2_ Laser to Treat Surgical Scars

**FIGURE 3:**
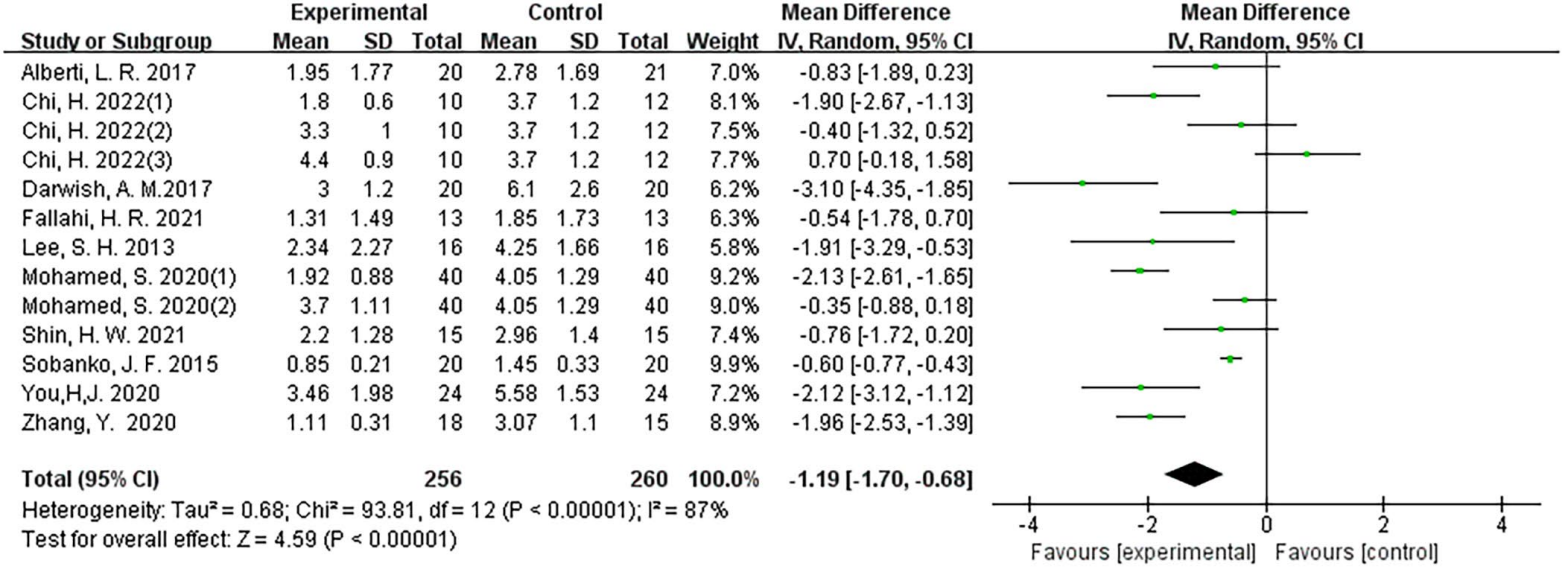
Forest graphs showing VSS score differences in RCT and Non‐RCT.

**FIGURE 4:**
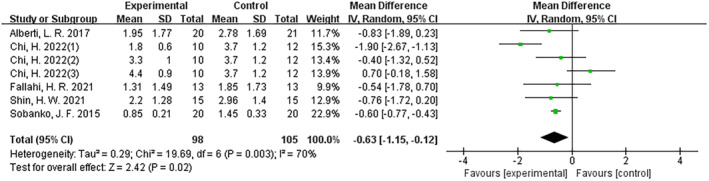
Forest graphs showing VSS score differences in RCT.

**FIGURE 5:**
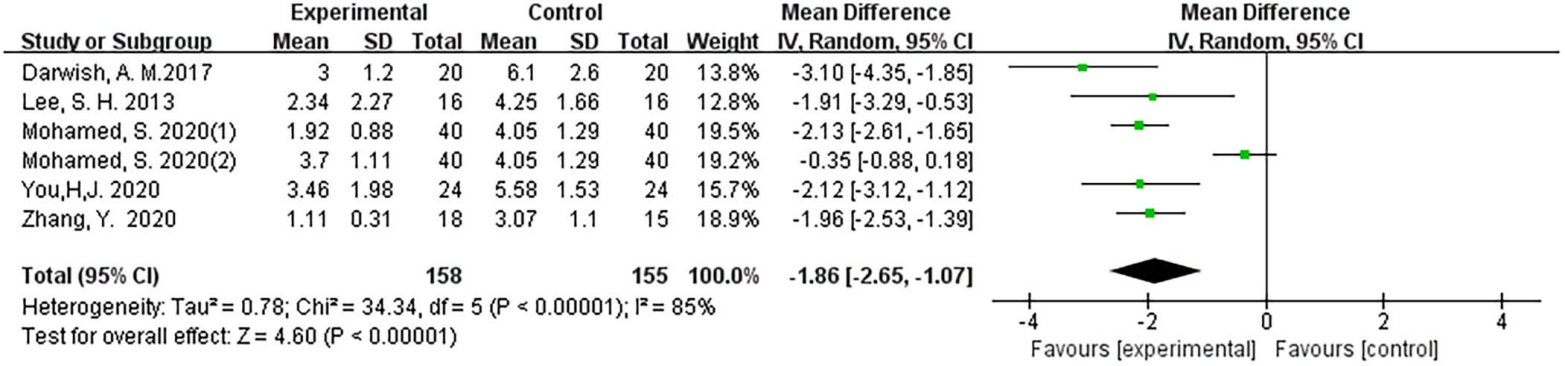
Forest graphs showing VSS score differences in non‐RCT.

The results demonstrated the efficacy of fractional CO_2_ laser therapy across all controlled trials (mean difference (MD): –1.19; 95% CI: −1.70 to −0.68; I^2^ = 87%; *p* < 0.001) (Figure 3). Both in RCT and non‐RCT settings, fractional CO_2_ laser therapy exhibited the same efficacious outcomes, with MD values of −0.63 (95% CI: −1.15 to −0.12; I2 = 70%; *p* = 0.02) and − 1.86 (95% CI: −2.65 to −1.07; I2 = 85%; *p* < 0.001), respectively (Figures 4, 5).

### 3.5 Meta‐Analysis Results on Optimal Timing for Treating Surgical Scars

**FIGURE 6:**
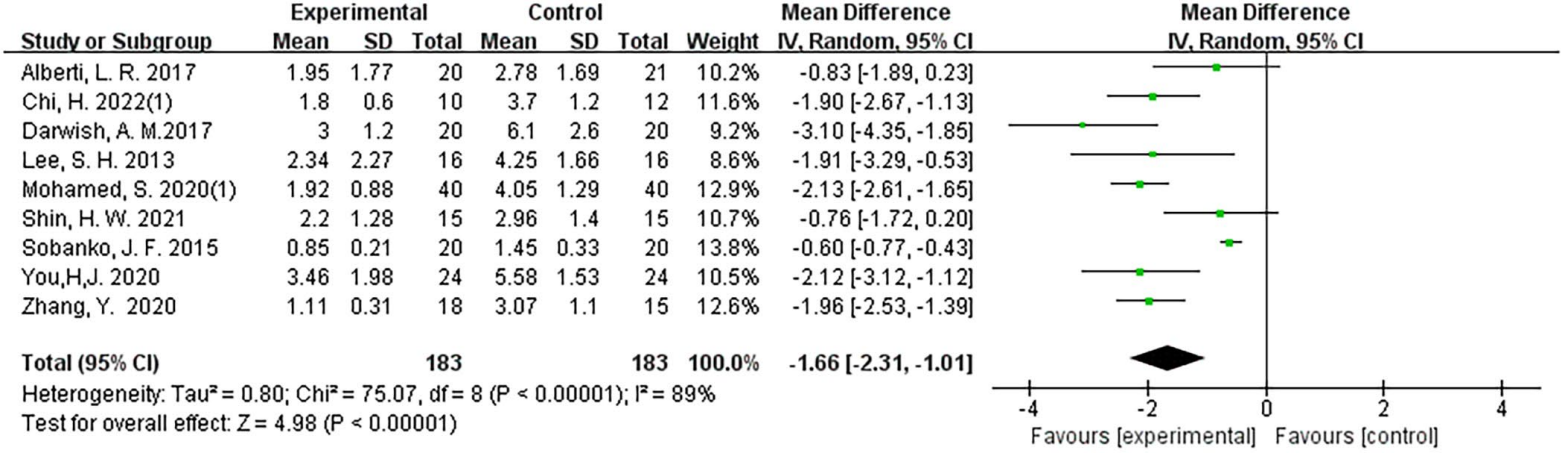
Forest graphs showing VSS score differences for group starting time at or within 1 month after surgery versus control group.

**FIGURE 7:**
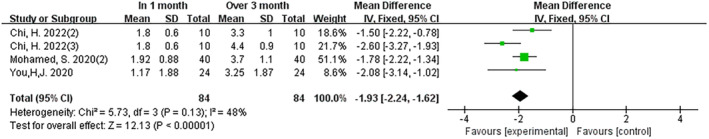
Forest graphs showing VSS score differences for starting time at or within 1 month after surgery versus more than 3 months after surgery.

**FIGURE 8:**
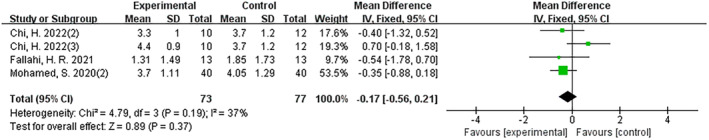
Forest graphs showing VSS score differences for group starting time more than 3 months after surgery versus control group.

**FIGURE 9:**
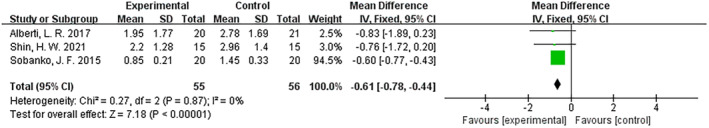
Forest graphs showing VSS score differences for starting time within 1 month after surgery and only preformed a single session.

The study analysis showed that initiating fractional CO_2_ laser treatment sessions at or within 1 month after surgery significantly reduced postoperative scars compared to control groups and groups on treatments initiated more than 3 months after surgery (MD: −1.66; 95% CI: −2.31 to −1.01; I2 = 89%; *p* < 0.001 and MD: −1.93; 95% CI: −2.24 to −1.62; I2 = 48%; *p* < 0.001) (Figures 6, 7). However, fractional CO_2_ laser treatment administered over 3 months after surgery did not significantly improve postoperative scars (MD: –0.17; 95% CI: −0.56 to 0.21; I2 = 37%; *p* = 0.37) (Figure 8). This study also found that completing only one treatment session within 1 month after surgery can produce significant results (MD: –0.61; 95% CI: −0.78 to 0.44; I2 = 0%; *p* < 0.001) (Figure 9).

### 3.6 Sensitivity Analysis and Publication Bias

**FIGURE 10:**
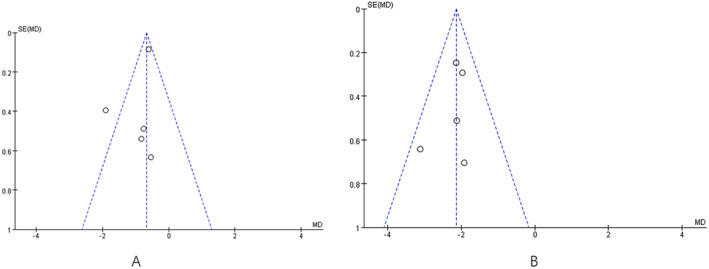
Funnel graphs of the meta‐analysis. (A) Funnel graph of the meta‐analysis for RCTs, (B) Funnel graph of the meta‐analysis for N‐RCTs. SE (log [OR]), the standard error of the logarithm of the effect size. OR, odds ratio; SE, standard error.

Based on the analysis of sensitivity and publication bias, illustrated in Figure 10, it is apparent that there is asymmetry in the funnel plot for both RCTs and N‐RCTs included in this meta‐analysis. This suggests the possibility of publication bias influencing the assessment of fractional CO_2_ laser treatment effectiveness for surgical scars.

## 4 Discussion

The traditional CO_2_ laser can result in the vaporization of the epidermis and coupled with the thermal damage it influences collagen denaturation and contraction, which will be similar to dermabrasion and can make skin repair and reconstruction [28]. Therefore, this laser is widely used to improve functions and cosmetics of burn scars [21, 22, 29, 30, 31]. However, large‐scale treatment easily produces color sink and detachment. When it acts on the skin under the premise of ensuring enough penetration depth, fractional CO_2_ laser can not only regulate the lattice density, but also regulate the penetration depth. The heat of adjacent tissue can diffuse in time, and will not form heat accumulation, which reduces heat damage and decreases the possibility of various complications. The studies on treating surgical scars were beginning to be gradually published. In this study, we also found fractional CO_2_ laser therapy to be an effective method for surgical scars.

The standard wound healing process consists of three overlapping phases: hemostasis/inflammatory phase (lasting 4–6 days), proliferative phase (from day 4 to 21), and remodeling phase (from day 21 to 2 years). Wounds in the proliferative phase often appear red, slightly raised, and may be accompanied by subtle itching and pain. They develop into mature scars within months [32]. Since re‐epithelialization after surgery is usually complete by weeks 2–3, and scars are still immature within 1–3 weeks of suture removal, initiating treatment at this stage has been shown to yield positive outcomes in most trials. In our study, 10 treatment trials were completed at or within 1 month after surgery. Although they demonstrate the beneficial effects of fractional CO_2_ laser treatments in three consecutive treatments of mature caesarean scars, Karmisholt et al. still support that early fractional CO_2_ laser intervention after surgery might garner superior scar outcomes [17] Studies on fractional CO_2_ laser for cleft lip scars by Mohamed Shada et al. and Chi, H. et al. also support early fractional CO_2_ laser application, at 1 month after surgery or within the first 3 weeks after repairing, showing significantly better results compared to delayed treatment at 3 months post‐surgery [22, 25]. Similarly, You, H. J et al. observed greater improvement in thyroidectomy scars with early fractional CO_2_ laser treatment compared to untreated or late‐treated controls[21]. This review and meta‐analysis provide evidence that fractional CO2 laser treatment is most effective within 1 month after surgery. Although no statistically significant differences in improvement were observed when comparing treatments administered at control groups and more than 3 months groups after surgery, the overall trend suggests that treatments performed after 3 months may be less effective. The research by Lin, M. J. et al. further suggests that early intraoperative CO_2_ laser treatment is not inferior to CO_2_ laser treatment performed on postoperative scars on the 14th day [27]. However, the timing of laser treatment should be individualized, taking into account the specific circumstances of the patient.

Sobanko, J.F. et al. suggest that a single fractional CO_2_ laser treatment at 1 week after surgery may not significantly impact facial scar appearance long‐term, and multiple treatment sessions could yield greater results [16]. Most patients in reviewed studies underwent two or three fractional CO_2_ laser treatments, with You, H.J et al. completing five treatments. However, meta‐analysis indicates that even one treatment within a month post‐surgery can yield significant results [21]. In this review, there is also an experiment on comparing energy levels, a comparison of energy levels by Wang, J. et al. did not reveal differences in scar appearance between high fluences with low density versus low fluences with low density of fractional CO_2_ laser treatment [26].

Currently, there are numerous scar evaluation scales, but no universal standard. Many trials suggest fractional CO_2_ laser intervention on surgical wounds achieves favorable outcomes with VSS scores. However, Buelens, S. noted that the POSAS failed to detect objective differences, and objective measurements could not confirm its efficacy in treating recent surgical scars, because the POSAS considers subjective symptoms and patient opinion [14]. The POSAS allows taking into account any subjective symptoms and the patients' opinions [33].

Karmisholt et al., utilizing both VSS and POSAS, investigated the value of both scoring systems but focused on mature caesarean scars [17]. Trials treating immature scars within 1–3 weeks post‐suture removal using both scoring systems are warranted. You, H.J. et al.'s research demonstrated significantly higher mean PGA scores for early‐treated scar halves at 6th and 11th month follow‐ups [21]. However, further literature on these scoring systems is needed.

There are several limitations in this study, First, there are fewer randomized controlled trials and blinding of participants and personnel was also in only five trials. Second, the lack of concealment of the patient might have influenced a placebo effect. Third, these controlled studies This study did not find comparative trials between a single treatment course and multiple treatment courses, and therefore relevant experimental studies are required.

## 5 Conclusion

The systematic review and meta‐analysis provide support for the efficacy of fractional CO_2_ laser treatment for surgical scars, particularly when administered during surgery or within 1 month postoperatively. Although a single treatment within the first month can produce significant effects, most clinical trials support the use of 2–3 or more treatments. However, it is important to note that the current body of evidence is limited by the lack of RCT trials, highlighting the need for further high‐quality research to validate these findings.

## Author Contributions

Qiang Ji, MD, carried out the development or design of the methodology, statistical analysis, and original draft. Lili Luo MS helped critically revise the draft and data curation. Lili Luo, MS. is the co‐first author. Jun Ni, Xiaolan Pu, and He Qiu, MD. participated in the methodology, data curation, and validation. Dongmei Wu, MD. designed the conceptualization and carried out the article and editing. All authors have checked and approved the final manuscript.

## Conflicts of Interest

The authors declare no conflicts of interest.

## Human and Animal Rights

This article does not contain any studies with human participants or animals performed by any of the authors. Informed Consent For this type of study, informed consent is not required.

## Data Availability

The data that support the findings of this study are available on request from the corresponding author. The data are not publicly available due to privacy or ethical restrictions.
